# Comparison of PSA density and lesion volume strategies for selecting men with equivocal PI-RADS 3 lesions on bpMRI for biopsies

**DOI:** 10.1007/s00261-022-03720-0

**Published:** 2022-11-01

**Authors:** Karen-Cecilie Kortenbach, Vibeke Løgager, Henrik S. Thomsen, Lars Boesen

**Affiliations:** 1 Department of Radiology, Herlev Gentofte University Hospital, Borgmester Ib Juuls Vej 17, 2730 Herlev, Denmark; 2 Department of Urological Research, Herlev Gentofte University Hospital, Herlev, Denmark

**Keywords:** Biparametric MRI, Multiparametric MRI, Prostate cancer, Equivocal PI-RADS 3, TRUS biopsy

## Abstract

**Purpose:**

To compare two strategies: Prostate-specific antigen density (PSAd) and lesion volume measurement in ruling out significant prostate cancer (sPCa) in men with equivocal Prostate Imaging Reporting and Data System (PI-RADS) category 3 index lesions on biparametric magnetic resonance imaging.

**Methods:**

In total, 130 men from our database had index lesions with PI-RADS scores of 3. Prostate volume was measured using the ellipsoid method, in accordance with PI-RADS version 2.1 criteria. Index lesion volumes were also measured using the ellipsoidal formula on the diffusion-weighted imaging sequence with the highest *b*-value and sagittal T2 sequences.

**Results:**

Among 130 men with PI-RADS category 3 index lesions, 23 (18%) had sPCa. In total, 6 of the 89 men with PSAd < 0.15 ng/mL^2^ (7%) had sPCa, whereas 8 of the 49 men with index lesion volumes < 0.5 mL (16%) had sPCa. The difference was statistically significant (McNemar, *p* < 0.0001).

**Conclusion:**

The PSAd strategy performed better than the lesion volume strategy in ruling out sPCa in men with equivocal PI-RADS category 3 index lesions.

## Introduction

**Fig. 1:**
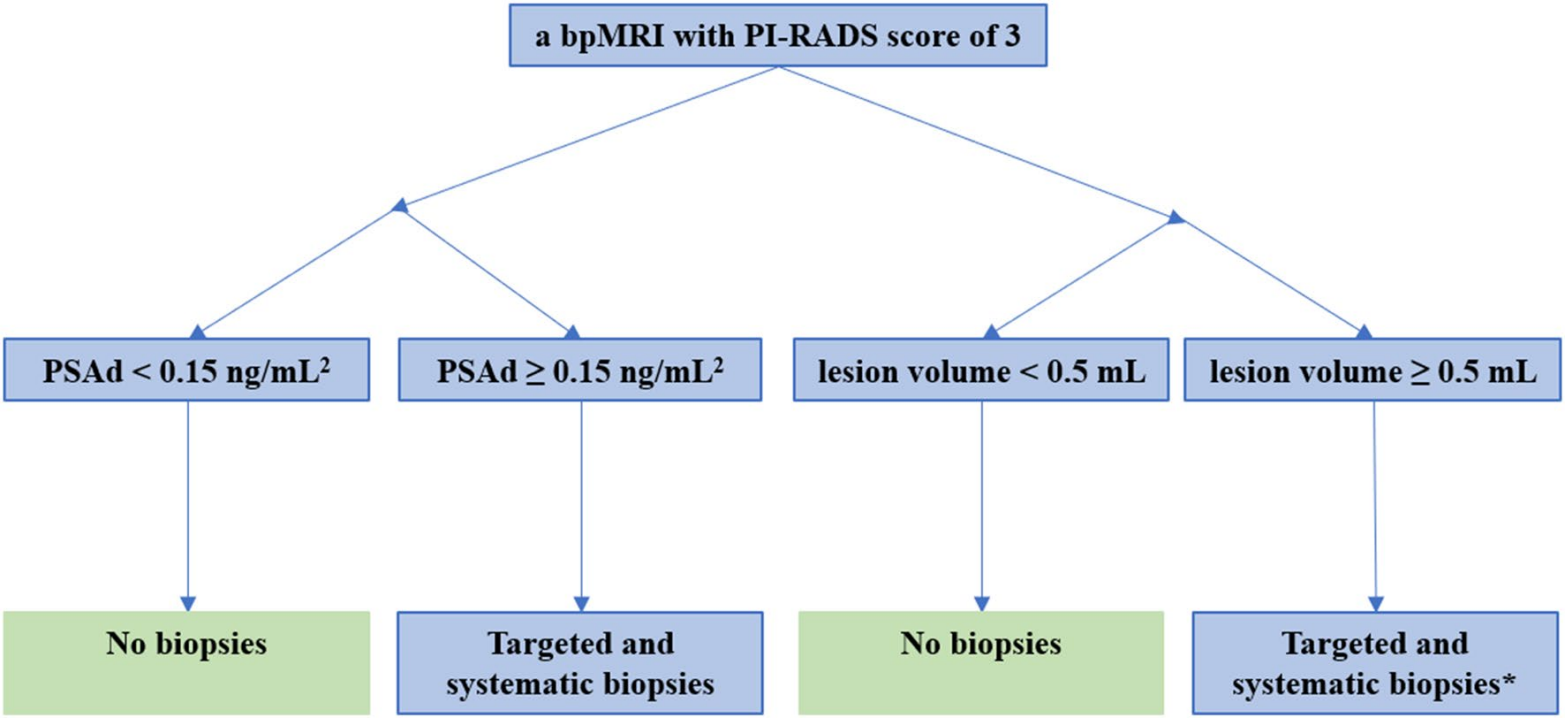
The two strategies * Only targeted biopsies are recommended by Scialpi et al., The strategy is a proposed strategy as the study population had systematic biopsies too, because this was a retrospective study [20]. Abbreviations: *PSAd* prostate-specific antigen density, *bpMRI* biparametric magnetic resonance imaging, *PI-RADS* Prostate Imaging Reporting and Data System. SI conversion factor: To convert PSAd to micrograms per liter squared, multiply by 1.0

Biparametric (bp) magnetic resonance imaging (MRI) is increasingly used for prostate imaging because it results in similar significant (s) prostate cancer (PCa) detection rates compared to the more time consuming and costly multiparametric (mp) MRI [1–5]. However, there is no consensus on how to report bpMRI results in this context. The lack of a universal scoring system and standardized risk assessment procedures has hampered implementation of bpMRI as an alternative to mpMRI. The Prostate Imaging Reporting and Data System (PI-RADS) committee has requested more evidence before they can create a reporting system and guide to using PI-RADS with bpMRI [6]. Because bpMRI does not use contrast media, this procedure may be less effective for characterizing equivocal PI-RADS category 3 lesions. If the clinical priority is to avoid missing sPCa, biopsies should be recommended for these lesions, but this will result in more unnecessary biopsies and perhaps overdiagnosis of insignificant PCa [5]. One way to solve this problem involves combining bpMRI scores with other biomarkers such as prostate-specific antigen density (PSAd) [7, 8]. Unfortunately, both PCa and conditions that frequently affect older men, such as benign prostatic hypertrophy, prostatitis, and urinary retention can alter PSA levels in the blood [9, 10]. Thus, PSA is organ specific, but unfortunately it is not a PCa-specific marker. Consequently, the parameter PSAd was introduced to better differentiate between benign and malignant causes of PSA elevation. PSAd is calculated by dividing the PSA level by the volume of the prostate to allow for the potential influence of benign prostatic hypertrophy. Using PSAd for risk stratification often entails applying a PSAd cut-off of 0.15 ng/mL^2^, which may be used to separate men with PI-RADS 3 findings into two risk categories and determine whether biopsies should be offered (Fig. 1). This strategy is based on previously published studies [5, 8] and the European Association of Urology guidelines [11]. Another approach, suggested by Scialpi et al., combines the volume of the index lesion (cut-off, 0.5 mL) and bpMRI scores to stratify risk and determine whether biopsies should be offered (Fig. 1) [12]. The two strategies differ as PSAd is an organ-based biomarker and is influenced by the entire prostate, whereas lesion volume depends only on a particular lesion and its size.

The purpose of this study is to compare the accuracy of PSAd and lesion volume measurements in ruling out sPCa in men with equivocal PI-RADS category 3 index lesions on bpMRI.

## Materials and methods

**Fig. 2:**
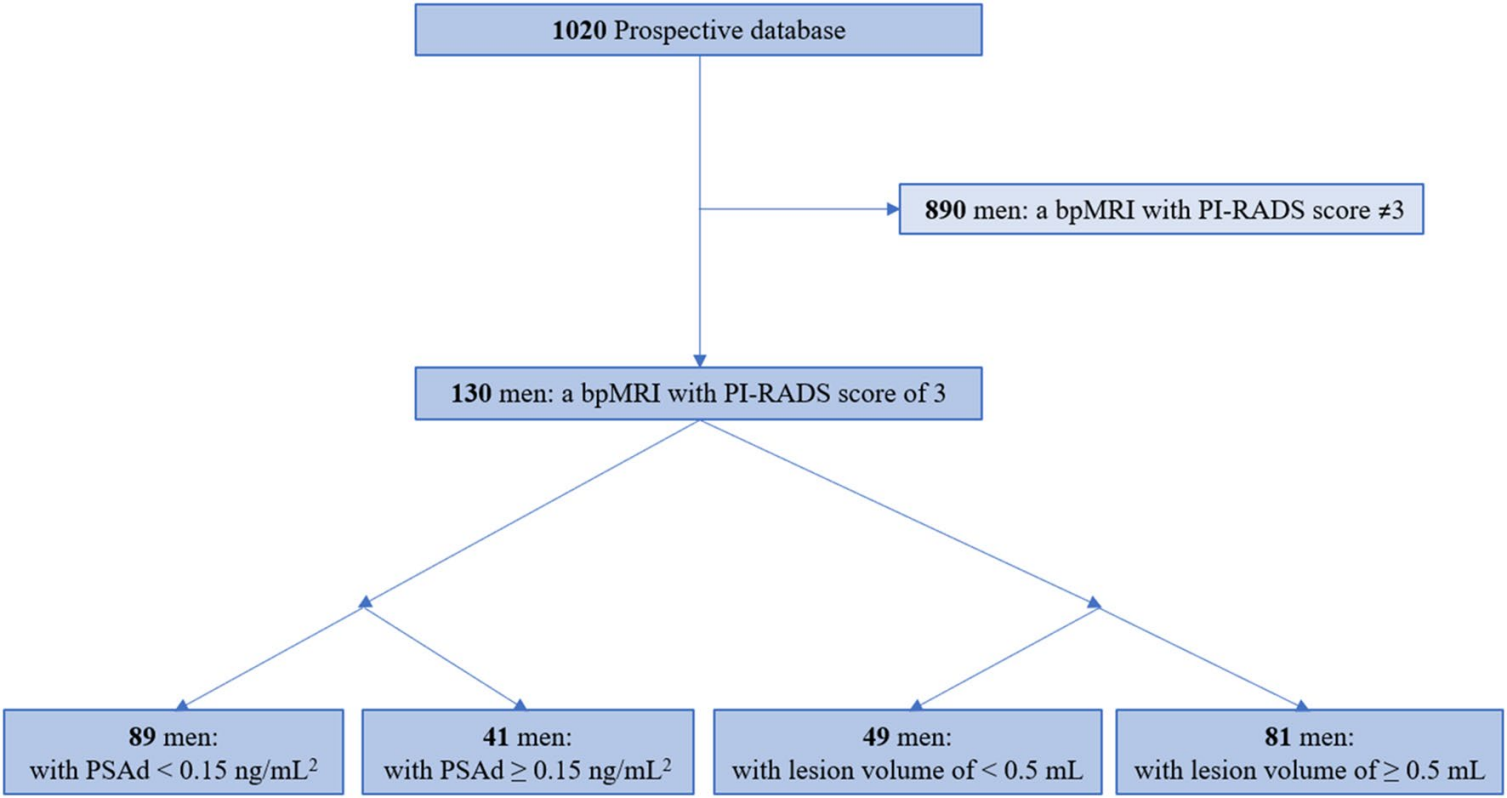
Men excluded from study Abbreviations: *PSAd* prostate-specific antigen density, *bpMRI* biparametric magnetic resonance imaging, *PI-RADS* Prostate Imaging Reporting and Data System. SI conversion factor: To convert PSAd to micrograms per liter squared, multiply by 1.0

The study population was a retrospective cohort, derived from the prospective database that the Biparametric MRI for Detection of Prostate Cancer (BIDOC) study used to assess the diagnostic accuracy of bpMRI for PCa detection [5]. The BIDOC database included 1020 biopsy-naïve men with clinical suspicion of PCa who underwent bpMRI followed by transrectal ultrasound-guided biopsies with systematic standard biopsies and MRI/transrectal ultrasound fusion-targeted biopsies of any lesion with a PI-RADS score ≥ 3 during the period November 2015 to June 2017. Of these, 130 men had index lesions with PI-RADS scores of 3 (Fig. 2). Prostate volume was measured using the ellipsoid method, in accordance with PI-RADS version 2.1 criteria [13]. Index lesion volumes were also measured using the ellipsoidal formula on the diffusion-weighted imaging sequence with the highest *b*-value and sagittal T2 sequences [13]. The PSA density strategy and Index lesion volume strategy are proposed strategies and was not part of the original study strategy as all patients had biopsies.

### PSA density strategy

For PSAd, we used a cut-off of 0.15 ng/mL^2^ to separate men with PI-RADS 3 lesions into two categories. Men with PI-RADS 3 lesions and PSAd < 0.15 ng/mL^2^ avoid biopsies and are referred back to their general practitioner for clinical surveillance (i.e., routine PSA measurements and digital rectal examinations; Fig. 1). Conversely, men with PI-RADS 3 lesions and PSAd ≥ 0.15 ng/mL^2^ undergo both systematic and targeted biopsies of the PI-RADS 3 lesion (Fig. 1).

### Index lesion volume strategy

Scialpi et al. suggested using an index lesion volume cut-off value of 0.5 mL to separate men with PI-RADS 3 lesions into two categories (Fig. 1). Men with PI-RADS 3 index lesions that are < 0.5 mL in volume are not offered biopsies but are evaluated according to their age and clinical information, are followed-up by monitoring via PSA measurements, and are assessed by bpMRI within 1 year [12]. Men with PI-RADS 3 index lesions that are ≥ 0.5 mL in volume are recommended for targeted biopsies [12].

### Magnetic resonance imaging

Prior to biopsies, bpMRI examinations were performed using a 3-T MRI magnet (Ingenia version 5.3.1; Philips Healthcare, Best, the Netherlands) with a 16-channel surface coil and a built-in table coil (Philips Healthcare) positioned over the pelvis. The bpMRI protocol included a sagittal scout, axial T2-weighted and diffusion-weighted imaging (b-value 2000) with reconstructions of the corresponding apparent diffusion coefficient maps and image acquisition times of approximately 15 min. Imaging parameters are listed by Boesen et al. in Supplementary Table 1 [5]. All bpMRIs were reviewed by the same prostate MRI physician (5 years of experience), blinded to clinical findings. Any suspicious lesions were registered and scored on a 5-point scale according to their likelihood of being sPCa (1, highly unlikely; 2, unlikely; 3, equivocal; 4, likely; and 5, highly likely) using the PI-RADS version 2 criteria [14]. However, an equivocal score of 3 was not considered for upgrade to a score of 4, because the bpMRI protocol does not include dynamic contrast-enhanced imaging; thus, scoring of lesions in the peripheral zone relied solely on diffusion-weighted imaging findings (dominant sequence). Only men with bpMRIs showing a PI-RADS category 3 index lesion were included in this study (Fig. 2).

### Histological evaluations and cancer significance

The definition of sPCa was any core with PCa Gleason grade group (GG) ≥ 2. All biopsy samples were reviewed by the same genitourinary pathologist (> 15 years of experience). For each PCa-positive biopsy core, the location, Gleason score (GS) based on the International Society of Urological Pathology 2005 consensus, and percentage of cancerous tissue per core were determined [15]. In addition, men with tumor-containing biopsies were assigned to a GG in accordance with the International Society of Urological Pathology 2014 consensus guidelines [16].

### Statistical analysis

Patient characteristics are presented using descriptive statistics. Continuous variables (i.e., age, PSA level, PSAd, and prostate volume) are described using medians and interquartile ranges, and normality was tested using the Shapiro–Wilk method**.** Sensitivity, specificity, positive predictive value (PPV), and negative predictive value (NPV) were calculated using a 2 × 2 contingency table. Differences in false negative results between the two strategies were evaluated using McNemar's chi-squared test with a continuity correction. A *p*-value < 0.05 was considered statistically significant. Statistical analysis was performed using Rstudio software (ver. 1.1.5; RStudio, Inc., Boston, MA, USA) [17].

## Results

A total of 130 men with PI-RADS category 3 index lesions were retrospectively enrolled from our BIDOC database (Fig. 2). The study population had a median age of 64 years (interquartile range, 63–68 years), a median PSA level of 6.3 ng/mL (interquartile range, 5.2–9.1 ng/mL), a median prostate volume of 56 mL (interquartile range, 45–65 mL), and a median PSAd of 0.1 ng/mL^2^ (interquartile range, 0.1–0.2 ng/mL^2^). The data were not normally distributed (Shapiro–Wilk, *p*-value < 0.001). The distribution of men within each group is presented in Fig. 2.

Overall, sPCa was detected in 23 of the 130 men (18%) with PI-RADS category 3 index lesions. All 23 sPCa were detected by bpMRI targeted biopsies. In total, 7% (6/89) of the men with PSAd < 0.15 ng/mL^2^ had sPCa, whereas 16% (8/49) of the men with index lesion volumes < 0.5 mL had sPCa (Table 1). The difference was statistically significant (McNemar, *p* < 0.001). The distribution of biopsy results is presented in Table 1.

**Table 1:** Biopsy results from each group

Number (%)	PSA density		Lesion volume	
	≥ 0.15 ng/mL^2^	< 0.15 ng/mL^2^	≥ 0.5 mL	< 0.5 mL
no PCa	13 (32%)	56 (63%)	44 (54%)	25 (51%)
GG 1	11 (27%)	27 (30%)	22 (27%)	16 (33%)
GG 2	14 (34%)	4 (5%)	11 (14%)	7 (14%)
GG 3	1 (2%)	2 (2%)	3 (4%)	0 (0%)
GG 4	2 (5%)	0 (0%)	1 (1%)	1 (2%)
GG 5	0 (0%)	0 (0%)	0 (0%)	0 (0%)
**Total**	41 (100%)	89 (100%)	81 (100%)	49 (100%)

*GG* Gleason grade group, *PCa* prostate cancer, *PSA* prostate-specific antigen. SI conversion factor: To convert PSAd to micrograms per liter squared, multiply by 1.0

For the PSAd strategy, sensitivity was 0.74 and specificity was 0.78; for the index lesion volume strategy, sensitivity was 0.65 and specificity was 0.38 (Table 2). For the PSAd strategy, the PPV was 0.42 and the NPV was 0.93; for the index lesion volume strategy, the PPV was 0.19 and the NPV was 0.83 (Table 2). The 2 × 2 contingency table for the strategies is presented (Table 2).

**Table 2:** 2 × 2 contingency table for the two strategies

	Positives		Negatives	
	(GG 2, 3, 4, and 5)		(No PCa and GG 1)	
	PSA density	Lesion volume	PSA density	Lesion volume
True	17	15	83	41
False	24	66	6	8
**Total**	41	81	89	49

*PCa* prostate cancer, *GG* Gleason grade group, *PSA* prostate-specific antigen

## Discussion

When the two strategies for avoiding biopsies were compared, the index lesion volume strategy resulted in a significantly greater proportion of missed sPCas than the PSAd strategy (16% vs. 7%). Furthermore, two men had GG 4 PCa detected. Both of these men had PSAd ≥ 0.15 ng/mL^2^ and would have been recommended for biopsies, but only one had an index lesion volume ≥ 0.5 mL (Table 1). Consequently, one man with high-grade GG 4 disease would have been missed if the lesion volume strategy alone had been used to recommend biopsies.

The PSAd strategy had a higher NPV than the index lesion volume strategy for ruling out sPCa (93% vs. 83%). Consequently, the PSAs strategy is better at avoiding under-diagnosing of sPCa. However, the PPV of both strategies was low (42% vs. 19%).

The BIDOC study by Boesen et al. found that a prebiopsy bpMRI in biopsy naïve men had a low NPV for any PCa (72%) for a modified PI-RADS score of 3 or higher, but a high NPV for sPCa (97%) [5]. Boesen et al. defined sPCa as any core with high-grade PCa (GG ≥ 3) or a maximum cancerous core length greater than 50% of GG 2 PCa [5]. If the definition of sPCa was changed to any core with PCa GG ≥ 2, the NPV decreased to 93%.

Interestingly, a study of 141 men with PI-RADS 3 lesions on mpMRI was done to investigate whether PI-RADS 3 lesions changed over time [18]. Overall, 77% of men with PI-RADS 3 lesions exhibited a change from PI-RADS 3 to either PI-RADS 2 or 4 within the first year and 15% of all the patients harbored sPCa [18]. This observation suggests an additional strategy to the two evaluated in our study. Instead of immediate prostate biopsies, a repeat confirmatory mpMRI of men with PI-RADS 3 index lesions 1 year after the initial bpMRI may be beneficial, to discover whether any lesions have changed.

A recent study by Kortenbach et al. investigated 200 biopsy-naïve men with clinical suspicion of PCa who underwent a prebiopsy bpMRI and had PSAd measured, with a 2-year clinical follow-up [20]. The same PSAd strategy was applied in that study as we used in the present study: If a man had a PI-RADS score of 3 and a calculated PSAd < 0.15 ng/mL^2^, no biopsies were performed. If the man had a PSAd ≥ 0.15 ng/mL^2^ then he had targeted and systematic biopsies. Among 109 men with a PI-RADS score of 1–3, 11 men (10%) had a PI-RADS 3 lesion, distributed as four men with PSAd < 0.15 ng/mL^2^ and seven men with a PSAd ≥ 0.15 ng/mL^2^. The biopsies from the men with PSAd ≥ 0.15 ng/mL^2^ revealed that five men had sPCa and two men had no cancer [19]. The four men with PSAd < 0.15 ng/mL^2^ had no biopsies initially and no further biopsies during the follow-up surveillance period, which involved monitoring PSA levels, follow-up multiparametric MRI and digital rectal examinations [19]. The number of men with PI-RADS 3 lesions in the study by Kortenbach et al. is limited, but no man in that study who had a PI-RADS 3 lesion and PSAd < 0.15 ng/mL^2^ had sPCa detected within the follow-up period. This observation validates the results of our present study in which men with PI-RADS 3 lesions and PSAd < 0.15 ng/mL^2^ had a maximum GG of 3. However, in the study by Kortenbach et al., men with PI-RADS 3 lesions and PSAd < 0.15 ng/mL^2^ had no biopsies, which could have detected sPCas that were missed [19].

Studies have reported that tumors < 0.5 mL are unlikely to become clinically significant during a man’s life span and therefore do not warrant treatment [20, 21]. These studies support the index lesion volume strategy; however, approximately 20% of lesions are ≥ 0.5 mL and are more likely to develop to into sPCa. In addition, the approach recommended by Scialpi et al. uses MRI to measure lesion volumes and not prostatectomy specimens [12].

Both of the strategies compared in the present study could reduce unnecessary biopsies and increase the diagnostic yield of sPCa [7, 8, 20]. Neither strategy incurs additional costs. The PSAd strategy would not prolong reading times because we already use PSAd in our daily practice; therefore, the volume of every prostate is measured using MRI. We already measure lesion size, although not in three planes; however, this could be accommodated without substantially increasing reading times.

The main limitation of our study was the small study population. We would need a larger sample to reach more rigorous conclusions. However, the results we have warrant larger studies to validate these findings.

A second limitation was our study’s retrospective design. Unfortunately, bpMRI results were not read using the particular method recommended as part of the index lesion volume strategy. The retrospective design also means that this study is a sub analysis based on another study with a different purpose. Furthermore, although our study population had targeted biopsies, as recommended by Scialpi et al., they also had systematic biopsies, which were not recommended [12].

A third limitation is that we did not have prostatectomy-specimens as our reference test, which is a perfect gold standard. Our database was limited to the combined results of systematic and targeted biopsies. Thus, the final pathological results are unknown and sPCa could have been missed. This also means that we are unable to assess the true false negative rate.

Finally, no inter-reader assessments were performed as all bpMRI readings were reported by a single experienced urogenital radiologist. The performance of less experienced readers may affect the results as well as image quality and disease prevalence. When making clinical decisions based on MRI findings each institution should know their own test performance statistics.

In conclusion, the PSAd strategy performed better than the lesion volume strategy to rule out sPCa in men with equivocal PI-RADS category 3 index lesions, but larger studies are needed to support this conclusion.
